# Dupilumab therapy facilitates the histopathologic diagnosis of dermatomyositis in a patient with intercurrent severe atopic dermatitis and dermatomyositis

**DOI:** 10.1016/j.jdcr.2026.02.045

**Published:** 2026-03-06

**Authors:** Chris Wilson, Sara Castillo, Yvonne Ndoricimpa, Janine C. Malone, John Strickley

**Affiliations:** a University of Louisville School of Medicine, Louisville, Kentucky; b Division of Dermatology, Department of Medicine, University of Louisville School of Medicine, Louisville, Kentucky

**Keywords:** atopic dermatitis, case report, collagen vascular disease, dermatology, dermatomyositis, dermatopathology, dupilumab

## Introduction

Dupilumab is a monoclonal antibody that inhibits signal transductions by interleukin-4 and interleukin-13 in the T-helper2 pathway.^1^^,^^2^ It is commonly used to treat atopic dermatitis (AD), which is a chronic inflammatory skin disease mediated by T-helper2-associated cytokines.^3^^,^^4^ Dupilumab is not typically used to treat collagen vascular diseases (CVDs) and, in fact, a few cases of dupilumab-induced CVD or CVD-like eruptions have been reported.^1^, ^2^, ^3^ However, data regarding dupilumab’s role in these dermatoses varies, with 1 study reporting that it was not associated with autoimmune diseases such as rheumatoid arthritis and systemic lupus erythematosus (LE).^5^

The literature is remiss with therapeutic options for AD patients following inadequate response to dupilumab, and co-occurrent dermatoses should be considered.^4^^,^^6^ Furthermore, some studies suggest an association between AD and CVDs,^7^ however, there is currently no literature supporting co-occurrent dermatomyositis (DM) with AD and its management with dupilumab. DM is an idiopathic inflammatory myopathy characterized by distinct skin findings and skeletal muscle inflammation.^8^ To our knowledge, we report the first patient with intercurrent AD and DM whose DM was confirmed histopathologically following the resolution of AD with dupilumab therapy.

## CASE REPORT

**Fig 1:**
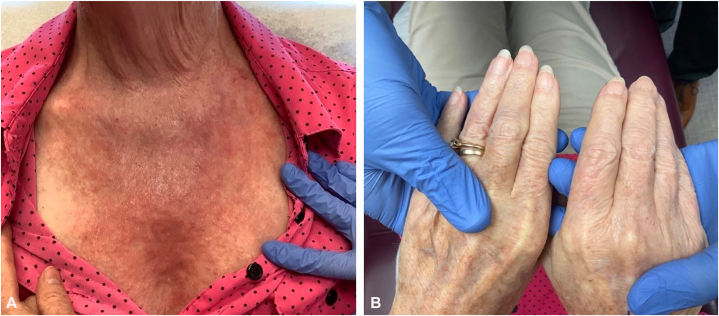
Clinical presentation demonstrated scaly, violaceous pruritic plaques with poikiloderma in shawl distribution **(A)** and ragged cuticles with periungual telangiectasia and capillary dropout **(B)**.

A 78-year-old woman with a history of nonmelanoma skin cancer presented with a pruritic eruption on the forearm, upper sternum, and back. On physical examination (Fig 1, *A* and *B*), she had scaly, pruritic plaques and violaceous erythema in shawl-like distribution on her upper sternum and back. Her cuticles were violaceous, ragged, and demonstrated hemorrhage and capillary dropout. In addition, she had scaly, eczematous and linear patches and plaques involving her chest, back, arms and legs. Physical examination was negative for facial edema, erythema, and holster sign. Initial labs were within normal limits, including complete blood count and comprehensive metabolic panel; aldolase, creatine kinase, and a full myositis panel were negative.

**Fig 2:**
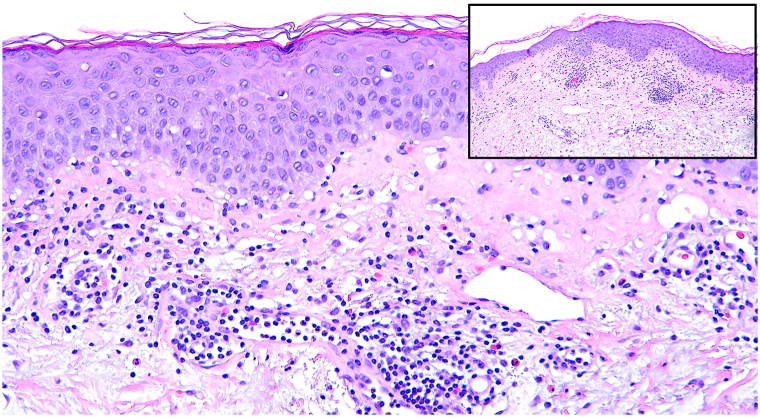
Initial H&E-stained biopsy (200X) of upper sternum demonstrating epidermal spongiosis, subtle basal vacuolar alteration and patchy superficial dermal inflammation including lymphocytes and many eosinophils; inset (100X) demonstrates spongiotic epidermal change and superficial inflammation.

A punch biopsy was taken from the upper sternum and histopathologic examination showed spongiosis with associated intraepidermal eosinophils, mixed superficial dermal inflammation with eosinophils, and subtle interface dermatitis (Fig 2). The composition of inflammation, predominantly eosinophils, did not support a diagnosis of DM and was most supportive of AD. The patient was subsequently treated with dupilumab (300 mg/2 mL).

**Fig 3:**
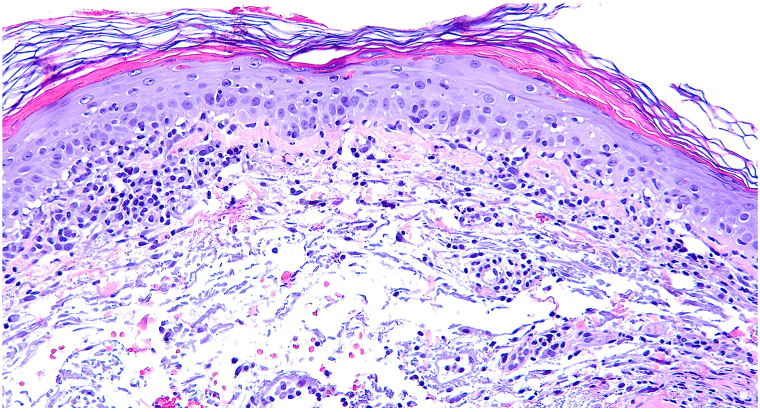
200X H&E: Final biopsy of upper sternum demonstrating vacuolar interface dermatitis associated with a mononuclear dermal inflammatory infiltrate.

The patient’s eruption improved significantly on dupilumab, though some pruritic areas on her scalp and neck persisted after 1 year of therapy. Interestingly, her cuticles initially improved with dupilumab, but subsequently became ragged with hemorrhage and capillary dropout after 1 year of treatment. She also exhibited significant muscle weakness upon standing. A repeat biopsy of her upper sternum demonstrated vacuolar interface dermatitis without eosinophils, indicative of a CVD such as DM (Fig 3). Repeat labs, including complete blood count and comprehensive metabolic panel were unchanged and within normal limits, however, a new myositis panel was not conducted at this time. The patient’s clinical and histopathologic findings were compatible with DM, and methotrexate was added to her treatment plan while continuing dupilumab. The patient was started on methotrexate (10 mg/wk) for 1 month and then increased to 15 mg/wk. Following 2 months of methotrexate therapy, her symptoms improved.

## Discussion

We present a patient with clinically apparent DM and concomitant AD who achieved a good clinical response of her AD to dupilumab, allowing us to appreciate her DM histologically. This clinical presentation differs from a few published cases where patients treated with dupilumab for AD were subsequently thought to have a drug-induced CVD-like reaction such as systemic LE, discoid LE, and subacute cutaneous LE.^1^, ^2^, ^3^ Our patient’s clinical findings of DM predated dupilumab therapy, and once the patient’s AD was cleared by the medication, the clinical features of DM persisted and became apparent histopathologically.

One possible explanation for our patient’s remarkable clinical response to dupilumab is that the AD may have exacerbated the inflammatory processes associated with DM. Some studies suggest a significant association between AD and CVDs, implying overlapping autoimmune mechanisms.^7^^,^^9^

Perhaps one of the most important considerations in this case is the dermatopathologic picture across the time-course of treatment. Initial biopsies showed subtle interface change with eosinophils consistent with AD, however, after initial treatment with dupilumab resolved the AD, a subsequent biopsy revealed well-developed interface dermatitis without eosinophils, compatible with a CVD. Alteration of the inflammatory milieu resulting from dupilumab therapy revealed diagnostic histomorphology of DM in a patient with co-occurrent AD and DM. Histopathologic evaluation was helpful in diagnosing this patient initially with AD and subsequently with underlying DM; and each diagnosis became clear based on the quantity of eosinophils noted pre- and post-treatment with dupilumab. The presence of eosinophils in biopsy specimens mitigates against a diagnosis of CVD. Sharon et al conducted a literature review regarding the diagnostic specificity of eosinophils in interface dermatitis, including 97 examples of interface dermatitis with clinically confirmed diagnoses. They found that eosinophils are rare to absent in LE, pityriasis lichenoides, graft-vs-host disease, and DM. Furthermore, their results suggest that the presence of a single eosinophil within 9 or 10 20X fields argues against a diagnosis of pityriasis lichenoides, DM, or LE.^10^

Identifying and treating co-occurrent dermatoses is challenging. Additionally, it is possible that the treatment of 1 dermatosis may reveal another dermatosis, and potential confounding dermatoses should be ruled out.^4^ Theoretically, if dupilumab doesn’t fully clear AD, another dermatosis must be considered.^4^^,^^6^ Bai et al reported 50 patients with incomplete response to dupilumab who were presumed to have AD. They found that all 50 patients with presumed AD and incomplete response to dupilumab had either concomitant conditions or entirely different diagnoses.^6^

In conclusion, we report a patient with intercurrent AD and DM whose DM diagnosis was confirmed following histopathologic evaluation of a biopsy taken after treatment with dupilumab despite a pre-treatment biopsy from the same anatomic site showing features inconsistent with DM. In so doing, we emphasize the importance of clinicopathologic correlation during/following therapy with medications which may alter clinical and histopathologic findings. Albeit rare, our case illustrates the importance of considering co-occurrent dermatoses based on clinical presentation and response to treatment and, more specifically, AD and a second dermatosis when dupilumab fails to resolve AD.

## Conflicts of interest

None disclosed.

## References

[1] Maeno M., Tamagawa-Mineoka R., Arakawa Y., Masuda K., Katoh N. (2022). Facial discoid lupus erythematosus during dupilumab treatment for atopic dermatitis. J Dermatol.

[2] Waugh M., Gavigan G. (2024). Drug-induced subacute cutaneous lupus erythematosus secondary to dupilumab: a case report. SAGE Open Med Case Rep.

[3] Okune M., Okiyama N., Fukuzono M., Sasaki K., Nomura T. (2022). Development of systemic lupus erythematosus after dupilumab treatment in a case of atopic dermatitis. J Dermatol.

[4] Narla S., Silverberg J.I., Simpson E.L. (2022). Management of inadequate response and adverse effects to dupilumab in atopic dermatitis. J Am Acad Dermatol.

[5] Bridgewood C., Wittmann M., Macleod T. (2022). T helper 2 IL-4/IL-13 dual blockade with dupilumab is linked to some emergent T helper 17‒Type diseases, including seronegative arthritis and enthesitis/enthesopathy, but not to humoral autoimmune diseases. J Invest Dermatol.

[6] Bai H., Murase E.M., Ashbaugh A.G., Botto N.B., Murase J.E. (2022). Diagnostic testing of eczematous dermatitis with incomplete response to dupilumab. J Am Acad Dermatol.

[7] Ponvilawan B., Charoenngam N., Wongtrakul W., Ungprasert P. (2021). Association of atopic dermatitis with an increased risk of systemic lupus erythematosus: a systematic review and meta-analysis. J Postgrad Med.

[8] Mainetti C., Terziroli Beretta-Piccoli B., Selmi C. (2017). Cutaneous manifestations of dermatomyositis: a comprehensive review. Clin Rev Allergy Immunol.

[9] Sekigawa I., Yoshiike T., Iida N., Hashimoto H., Ogawa H. (2003). Two cases of atopic dermatitis associated with autoimmune abnormalities. Rheumatology (Oxford).

[10] Sharon V.R., Koina T.H., Barr K.L., Fung M.A. (2012). Assessment of the 'no eosinophils' rule: are eosinophils truly absent in pityriasis lichenoides, connective tissue disease, and graft-vs.-host disease?. J Cutan Pathol.

